# Research on the Guiding Effect of CTCs on Postoperative Adjuvant Therapy for Patients with Early Stage Endometrial Cancer

**DOI:** 10.1155/2022/4327977

**Published:** 2022-05-31

**Authors:** Liguo Li, Huihui Zhai, Qiumei Zhang, Yuan Feng, Chunhui Yang, Hong Li, Hongfen He

**Affiliations:** Department of Gynaecology, The First People's Hospital of Qujing, Qujing, Yunnan, China

## Abstract

Endometrial tumor has increased in occurrence and fatality in China during the last 11 years, owing to inconsistent hormone use and modifications in people living surrounding and lifestyles. One of the three main gynaecological tumors is endometrial carcinoma (EC). Longer waiting duration of operation was linked to a lower chance of sustainability in endometrial tumor patients. Despite the great sustainability rate of endometrial tumor, only around 46 percent of patients undergo adjuvant treatment. Circulating tumor cells (CTCs), circulating tumor DNA (ctDNA), and circulating free DNA (cfDNA) are the most investigated tumor noninvasive indicators. These circulating biomarkers are important in the knowledge of metastasis and tumorigenesis, and they could help researchers comprehend how cancer dynamics evolve throughout the therapy and illness development. In patients with solid tumor, the existence of circulating tumor cells (CTCs) in the peripheral blood is linked to a weak prognosis. However, there is a scarcity of information on how to detect CTCs in endometrial cancer (EC). Hence, in this paper, we analyze the guiding effect of CTCs on postoperative adjuvant treatment for sufferers with initial phase endometrial tumor using multi-cox regression method. The dataset is selected and the blood samples are collected using plasma separation method. The CTC is detected using differential diagnosis. The morphology and biological features, Immunocytochemistry, Genomic analysis, Transcriptomic analysis, Proteomic analysis, and molecular analysis are performed and the outcomes are evaluated.

## 1. Introduction

In recent years, the way of life in developed countries has altered dramatically. Overweight was compounded by the absence of regular exercise and convenient access to the highest calorie items. Overweight is a very well known cause to a variety of disorders, particularly tumor. Internal secretory organ adipose tissue is a non-homogeneous organ. Several variations may be seen in it, such as the esterification of androgen to oestrogen, which impacts a greater prevalence of estrogen-related malignancies including breast and endometrial tumour because of their hyperfunction. Leptin, adiponectin, tumour necrosis factor, visfatin, resistin, omentin-1, and vaspin are among the active proteins released by adipose tissue [1].

Endometrial tumor is one of the most common malignant tumors of the upper reproductive tract, with an increasing prevalence and fatality rate. The number of Chinese women detected with Endometrial tumour has risen dramatically in the last decade. Furthermore, younger females have a greater disease frequency, and Endometrial cancer affects people of every ages, from young females to the elderly. Numerous threats for Endometrial tumor have been suggested, including overweight, age, and late menopause, but the exact process that produces the condition is still unknown. Presently, the surgery, radiation, and chemotherapy are the most common therapy for Endometrial tumor. Consequently, the total sustainability percentage of Endometrial tumor patients with developed phases is just 16% to 18% after 6 years. As a consequence, finding reliable detection and prognostic biomarkers as well as new therapeutic techniques for Endometrial tumor is critical [2].

Endometrial tumor, is the 4th most common gynaecologic tumor after breast, lung, and colorectal cancers, and has an important effect on women health all around the world. Because of the absence of proper detection prior to menopause, the incidence and prevalence of this disease has increased in recent years. In the meantime, because of the variations in lifestyles, patient's age at disease onset has reduced. On the basis of medical and histological features, endometrial tumor is divided into 2 categories. The most frequent type of endometrial tumor is Type I, also known as endometrioid adeno carcinoma. The next kind of endometrial cancer is Type II that has a greater frequency and a bad prognosis. Many researches are concentrating on precise molecular changes that assist in the initiation of endometrial malignancies, which are essential for efficient disease evaluation and treatment [3].

The typical indication of abnormal vaginal bleeding, as well as the subsequent thorough examinations, may be diagnosed and treated in the early stages. Furthermore, certain individuals have vague symptoms, and those with medium to elevated risk factors are still at increased threat of incidence, leading to a greater fatality rate [4].

Several circulating serum and plasma molecules have been proposed as potential biomarkers in the diagnosis of Endometrial carcinoma (EC). Neither of the suggested compounds, meanwhile, has been recognized as a marker for routine Endometrial tumor screening or detection. The high and consistently increasing prevalence of Endometrial tumor, the absence of progress in sustainability frequency, and the circumstance that Endometrial tumor is the major frequent malignancy of the women reproductive tract all motivated experimental studies intended to identify a screening and initial identification method in this tumor [5].

The circulating tumor cells guiding effect is analyzed and the postoperative adjuvant treatment for sufferers with initial phase of Endometrial cancer is analyzed in this research work. The further part of this article is structured as shown. Section II offers the literary works associated with this paper. Section III explains the proposed model. Section IV provides the performance analysis of the suggested method. And, finally, section V concludes the overall idea of the paper.

## 2. Related Works

The authors of [6] validated the ANXA2 expression in CTC from great risk of Endometrial Cancer sufferers. The cancer cell adherence of ANXA2 indicating CTC to the endothelium and localization for the development of micro-metastasis were then simulated in vitro and in vivo. Overexpression of ANXA2 does not include a benefit in the CTC attachment phase, but it could be relevant in later stages, imparting a higher migratory capability. Daunorubicin was identified as a prospective hit after researchers conducted a high-throughput screening (HTS) for molecules that particularly target ANXA2. Lastly, researchers demonstrated Daunorubicin's capability to limit the growth of ANXA2 overexpressing cancer cells in a three-dimensional transendothelial migration process as well as an in vivo system of late Endometrial Cancer. The authors of [7] showed that ctDNA evaluation might be used as a biomarker for initial identification recurrence detection and control. More research is required, therefore, to verify these discoveries and to investigate their possible effects on patient treatment. The authors of [8] studied to see if there was a link among CTC phenotypes and clinicopathological characteristics in people with initial cervical tumor. CTCs were separated and categorised. CTC numbers and morphologies were correlated with clinicopathological characteristics of sufferers. In phase I-IIA cervical tumor, CTCs with mesenchymal morphologies are linked to pelvic lymph node metastases and lymphatic vascular penetration. The identification of circulating tumor cell morphologies aids in the initial identification of cervical tumor micro-metastasis and illness state evaluation. The authors of [9] discussed the hopeful but still constrained progress of liquid biopsy in Endometrial tumor contrasted to another tumor including breast, colorectal, and prostate tumor. Latest evidence on circulating tumor material in reduced invasive blood samples, as well as alternative modalities of liquid biopsy including such uterine aspirates, is presented. Proteomic and genomic investigations on liquid-dependent uterine samples have yielded not only the best detection tools but also the most efficient techniques to deal with cancer heterogeneity. Similarly, circulating tumor cells (CTCs) and circulating tumor DNA (ctDNA) provide a possibility for accurate individual stratification, initial recurrent cancer assessment, and real-time surveillance of medication responses. The utility of liquid biopsy for accurate oncology in endometrial cancer will be evaluated by well-designed investigations and medical trials. The authors of [10] assessed the v-NOTES (transvaginal natural orifice transluminal endoscopic surgery) staging operation for severe obese individuals with initial phase type-1 endometrial tumor to see if it is accurate and efficient. Due to the growing danger of operational incidence and death that accompanies with raising BMI, developing protection operational methods to gynaecological diseases is critical. Researchers believe that v-NOTES is a protected, efficient, and feasible mildly extensive operation in highly overweighing individuals with initial endometrial tumor when conducted by a professional doctor, and that v-NOTES is a secure, efficient, and viable slightly extensive operation in excessive overweight people with early endometrial tumor. The authors of [11] examined the effects of adjunctive therapy, sociodemographic variables, and cancer characteristics on the longevity of individuals with non-metastatic simple cell endometrial tumor. In this group from the Norwegian Cancer Center Statistics, adjuvant treatment had no discernible impact on sustainability. Because of the dangerous nature of this illness, medical investigations are required to find the best adjuvant treatment for sufferers with non-metastasis in order to enhance medical results. The authors of [12] evaluated the link among nodal micro-metastases and endometrial tumor medical result is yet to be determined. Researchers examined 2393 individuals with endometrial tumor with and without nodal micro-metastases in a nationwide, registry- dependent investigation. Disease-free longevity was the key result metric. Endometrial tumor sufferers with nodal micro-metastases have a worse disease-free sustainable rate. Sufferers with micro-metastases who underwent adjuvant treatment had a better disorder-free sustainable rate. The authors of [13] analyzed the MMR method which was implemented to see if it could forecast endometrial tumor sufferers reaction to adjunctive treatment. The methylation state of MMR protein and MLH1 determines the reaction to adjunctive treatment in Endometrial tumor. The sufferers who are most likely to gain from adjunctive treatment could be identified using the MMR approach. The authors of [14] evaluated to see if our recent technique of chemotherapy with vaginal brachytherapy (VBT) is adequate, researchers examined at the results of grade IA serous endometrial malignancies managed at a single organization. Researchers provide one of the biggest groups of sufferers with phase IA serous Endometrial carcinoma. Our findings highlight the lower RFS and OS of sufferers with phase IA serous Endometrial tumor compared to those with endometrioid endometrial tumor. Although few institutions proceed to use EBRT for these sufferers, results indicate that radiotherapy restricted to VBT has a minimal pelvic recurrence frequency, as well as a significant systemic danger despite the therapy. Despite the unsatisfactory results in these sufferers, researchers recommend combining chemotherapy with brachytherapy. The authors of [15] assessed the sufferers with endometrial tumor, assessing the viability and identification frequencies of an SLN biopsy. The side-specific identification frequency, sensitivity, and false negative rate were all investigated. Patients with less than 51% myometrial invasion and less malignancies having undergone laparoscopy had greater identification rates and less false negatives. Depending on the results of the SLN biopsy, these sufferers may be able to avoid systemic lymphadenectomy. The authors of [16] analyzed the Micro-metastases (MIC) and separated cancer cells detected in the sentinel lymph glands of endometrial tumor sufferers. Researchers offer a meta-analysis of the documented literature to identify developments in postoperative care and the prevalence of MIC and ITCs following lymphatic mapping. However, after obtaining adjuvant treatment, sufferers with less volume metastases have a higher relative danger of recurrence, as per our observations. Since additional uterine variables are concerned in the prognosis, whether adjuvant treatment is essential becomes a point of contention. Multi-institutional cancer inventories may be able to offer some light on this crucial issue. The authors of [17] analyzed the advanced to medium and elevated danger of initial phase endometrial tumor, and researchers wanted to see if vaginal cuff brachytherapy and chemotherapy improves recurrence-free survival as matched to pelvic radiation treatment. There was no evidence that VCB/C was superior to pelvic RT. The acute toxicity of VCB/C was higher, while the delayed toxicity was equivalent. In elevated danger of initial phase endometrial carcinomas of every histology, pelvic RT only remains an efficient, well-tolerated, and appropriate adjuvant therapy. The authors of [18] evaluated using a large retrospective hospital-dependent registry, and comparing the 2 therapy regimens to see if there are any categories of sufferers who might gain from VBT + CT vs. EBRT. In sufferers with initial phase serous cancer, VBT + CT was linked to a better prognosis. Therapy approach was not related with a change in longevity for non-serous histology, though sufferers with elevated grade tumor and none of the nodal resection gained from EBRT. The authors of [19] evaluated how lymphadenectomy and adjuvant chemotherapy affected sufferers with symptoms, operation, and living standards. These therapy approaches, researchers expected, would have a significant impact on sufferers showed results at follow-up. Up to two years following adjuvant chemotherapy for endometrial tumor, sufferers showed significantly impaired operation and increased symptoms. Surgical phase did not appear to have a measurable effect on the wellbeing or symptoms in sufferer diagnosed solely by operations at follow-up. As a consequence, eliminating lymph nodes to personalize adjuvant treatment appears to be justifiable from the sufferer's perspective although attempts to identify replacements to standard chemotherapy should be accelerated. The authors of [20] assessed that the prognosis for elevated grade and non-endometrioid endometrial malignancies is unfavorable, and the absence of random eventual information has led to a diverse variety of adjuvant therapeutic procedure. The focus of this research was to see how various therapy regimens affected the results of sufferers with elevated danger in initial phase endometrial tumor. The centers' adjuvant therapeutic approaches varied, resulting in a diverse community. A contrast of phase I sufferers was made, classified by adjuvant therapy. To evaluate the frequency and sustainability among treatment groups, log-rank analyses and Cox proportional hazards methods were utilized. In this study, neither adjuvant radiation nor chemotherapy was linked to a better result in stage I, elevated danger in endometrial cancer [21].

## 3. Proposed Work

**Figure 1:**
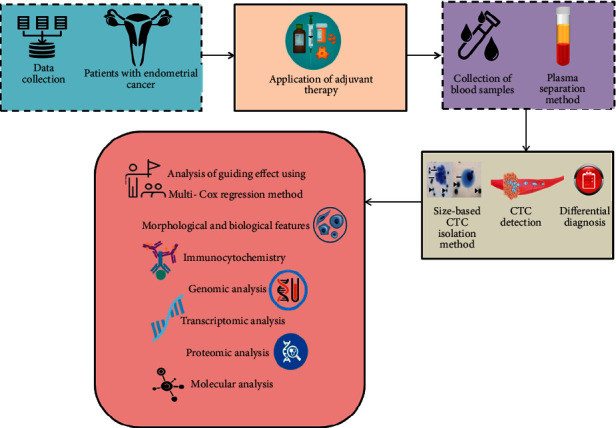
Schematic representation of the proposed method.

The patients' data with endometrial cancer is detected and the application of adjuvant treatment is analyzed and the blood samples are gathered for plasma separation method. Further, the differential diagnosis is performed by detecting the circulating tumor cells using size-based circulating tumor cell isolation method. The guiding effect using multi-cox regression method is employed for detecting the Endometrial cancer. The morphological and biological features such as immunocytochemistry, genomic, transcriptomic, proteomic, and molecular analysis of early endometrial cancer are analyzed.  Figure 1 shows the graphical depiction of the suggested method.

### 3.1. Data Collection

There were 4510 patients in total who had their clinical data gathered from The First People's Hospital of Qujing. There were 60 endometrial cancer instances and 1259 UF cases (age: 45.83 6.09 years) recognized, with 13 patients who presented with both EC and UF (age: 50.42 8.89 years). In the period between September 2014 and June 2020, patients were admitted to The First People's Hospital of Qujing. The hospital did color Doppler evaluations of the abdomen and vaginal passages prior to the surgery to ensure that everything was in order. Endometrial cancer and uterine fibroids (UFs) were diagnosed by pathological investigations. Patients who had had a caesarean section that was complicated by UFs were barred from participating. With a mean age of 43.98 years and a standard deviation of 9.64 years, there are 4537 patients in this study. The lowest age is 23 years.

### 3.2. Patient with Endometrial Cancer

The raw spectroscopic data was preprocessed after blood analysis to exclude non-biological interference that might have led to incorrect interpretation of the findings. The preprocessed spectra were then employed for multivariate analysis and class differentiation according to the final histopathology diagnosis, which was performed on the samples. Each patient had five successive spectra taken, for a total of 3260 spectra gathered from all of the patients. However, one study discovered that the development of plasma is related to the presence of electrons in the electron-hole pair. The researchers focused their attention on EC patients' mitochondrial DNA (mtDNA) copy number identification in peripheral blood leukocytes (PBLs) rather than serum or plasma in order to learn more about their condition. The presence of mutations in mitochondrial DNA may result in mitochondrial malfunction, which may contribute to tumor development. They found that having a low mtDNA copy number was associated with a five-fold rise in the chance of developing EC. They also designed a research to identify hypermethylation of the ZNF154 CpG island in five different kinds of tumors, including 42 EC patients, as part of their research. In addition to tissue samples, a computer simulation of ctDNA (1 percent tumor DNA in 99 percent normal DNA) was used in their investigation, and it was discovered that an AUC of 0.79 demonstrated the highest performance in endometrial cancers, which was confirmed by other researchers. The overall hysterectomy with bilateral salpingo-oophorectomy, para-aortic and pelvic lymphadenectomy, and pelvic washing to stage the illness are the mainstays of therapy for cervical cancer. There have been less postoperative problems related with laparoscopic surgery than with laparotomy, according to the literature. Due to the fact that it prohibits abdominal survey and lymphadenectomy, vaginal hysterectomy is often not indicated. Stage I carcinoma is a frequent kind of endometrial tumor seen in females. Surgical and histologic results determine whether or not additional treatment is required. Although lymphadenectomy of the pelvis and para-aortic lymph nodes is still debated, there have been some studies that have shown an improvement in survival as a result of the treatment, whereas others have found no such benefit. Patients that need lymph node staging are still being debated, and no agreement has been reached.

### 3.3. Application of Adjuvant Therapy

Despite the fact that postoperative radiation therapy may greatly lower the chance of local incidence, randomised viewpoint of studies in common have failed to show an improvement in overall survival in patients with initial phase of endometrial tumor. Most of the studies have not been powered for overall survival, and the competing threats of mortality in this senior group are substantial. However, the absence of an overall survival advantage has contributed to the continuing debate over the appropriateness of adjuvant therapy. Adjuvant treatment to the total pelvis might cause complications such as urine incontinence and faecal leakage, which can have a negative influence on quality of life for a lengthy period of time after the procedure. With the use of EBRT, one meta-analysis showed that minimum danger of sufferers with one-third or minimum MMI or intermediate one-third invasion with grade I or II disease had substantially inferior survival when compared to when they did not. The odds ratio for OS was 0.71 in this study. Patients with low-grade cancer who get radiation treatment do not have a shorter overall survival time. When administered in individuals with low-risk endometrial cancer, it has been linked to a decrease in quality of life as well as an increase in morbidity. Patients who are medically unable to undergo surgery might benefit from radiation treatment.

Adjuvant therapy should be avoided in sufferers who are at a low threat of developing cancer. For high threat of sufferers with initial phase of illness, vaginal brachytherapy (VBT) provides excellent local control at the vaginal cuff with reduced toxicity as compared to external beam radiation treatment (EBRT), but does not provide complete pelvic coverage. Sufferers with locally advanced tumor are often treated with external beam radiation therapy (EBRT) to encompass nodal areas at risk. The choice to employ adjuvant radiation and the precise treatments used (VBT and/or EBRT) may be contentious, and the decision is often based on a physician's evaluation of individual clinicopathologic characteristics, whether lymph node biopsy was conducted, and if chemotherapy was used.

### 3.4. Collection of Blood Samples

All instances of endometriosis were categorised using the new American Society for Reproductive Medicine scoring method, which was recently updated. A collection of fasting venous blood (5 ml, EDTA) was taken 1 day prior to the procedure from the antecubital vein and kept at 4 degrees of Celsius for a maximum of one hour before being used for further processing. The blood was centrifuged for 15 minutes at 1800 *g* at 19 degrees of Celsius, and the plasma was extracted. Once the plasma samples were aliquoted, they were kept in the refrigerator at 80 degrees of Celsius until further analysis.

### 3.5. Plasma Separation

Tubes containing ethylene diamine tetra acetic acid (EDTA) were used to collect blood samples from EC (Endometrial cancer) patients and healthy volunteers. Blood samples were sorted into cell-free plasma within 6 hours after being drawn from the patient. Using a two-step centrifugation process (350 reactive centrifugal force (RCF) for 10 minutes at 4 degrees of Celsius, followed by 20,000 RCF for 10 minutes), the plasma samples were separated and stored at 80 degrees of Celsius. EC tissue specimens and surrounding normal tissue samples were acquired from 24 EC patients who did not receive preoperative chemoradiotherapy and were processed in a formalin-fixed paraffin-embedded (FFPE) format.

### 3.6. Differential Diagnosis Using CTC Detection

Each patient had a total of 6 mL of peripheral blood drawn in the morning before surgery, which was then processed. CTCs were isolated utilizing an optimised CTC enrichment approach, which was done with the help of the CD45 antibody. In the first step, erythrocytes were eliminated via the use of red blood cell lysis, and then CD45+ leukocytes were decreased through the use of magnetic bead separation. In this study, CTCs were enhanced by passing them through calibrated membrane filters with 8-micron-diameter holes, and CTCs were detected and described using RNA-in situ hybridization (ISH). According to previously described branched DNA (bDNA) signal amplification method, CTCs were categorised utilizing EMT markers into 3 subpopulations: epithelial CTCs (CTCs that expressed the epithelial marker EpCAM or CK8), mesenchymal CTCs, and combined phenotypic CTCs (CTCs that expressed both epithelial and mesenchymal. The detection of cell searches was also carried out.

### 3.7. Size-Based CTC Isolation Method

In this case, cell size is important since CTCs are often larger (9–19 *μ*m) than normal blood cells (which are around 8 *μ*m in size). In order to minimise mechanical damage to cells during filtering, size-dependent approaches use membrane microfilters and microfluidics under controlled pressure. This technique eliminates the inaccuracies associated with diverse antigen expression reported in CTCs. There is a significant benefit to using this strategy since it does not need the use of labels, for example ISET, ScreenCellCyto, and Parasortix.

### 3.8. Analysis of Guiding Effect Using Multi-Cox Regression Model

Researchers found *m* distinct and unfiltered survival times ta (1 ≤ *a* ≤ *m*) in a sample of *n* people between the *n* conceivably censored survival times ti (1 ≤ *i* ≤ *n*). Each person is associated with a covariate vector *xi *=* *(*xi1*,. . ., *xir*,. . ., *xik*), as well as a censoring indication *i*. (0 denoted dead, 1 denoted censored). *R*_*a*_ refers to the group of people who were living and uncensored before to ta, also known as the risk set.

(1) $$\begin{array}{c} \text{log}S\left(\beta \right)=\sum_{a=1}^{n}\left[o_{a}\beta -\text{log}\left\{\sum_{h\in R_{a}}\text{exp}\left(o_{h}\beta \right)\right\}\right]=\sum_{a=1}^{n}S_{a}, \end{array}$$

where *S*_*a*_ impact to the log probability at the moment of failure *t*_*a*_. Taking the Initial derivatives of log L (*β*) and setting them,

(2) $$\begin{array}{c} \frac{\partial \text{log}S\left(\beta \right)}{\partial \beta_{r}}=\sum_{a=1}^{n}\left[o_{ar}-\frac{\sum_{h\in R_{a}}o_{hr}\text{exp}\left(o_{h}\beta \right)}{\sum_{h\in R_{a}}\text{exp}\left(o_{h}\beta \right)}\right]=\sum_{a=1}^{n}\frac{\partial s_{a}}{\partial \beta_{r}},\;1\leq r\leq k. \end{array}$$

When a function is reduced to zero *β*, it produces estimates of that must be determined by iteration, often utilizing the Newton–Raphson approach. The following are the elements of the associated information matrix I:

(3) $$\begin{array}{c} I_{rs}=\frac{-\partial^{2}\text{log}S\left(\beta \right)}{\partial \beta_{r}\partial \beta_{s}}=\sum_{a=1}^{n}\frac{-\partial^{2}s_{a}}{\partial \beta_{r}\partial \beta_{s}}=\sum_{a=1}^{n}\left\{\frac{\sum_{h\in R_{a}}o_{hr}o_{hs}\text{exp}\left(o_{h}\beta \right)}{\sum_{h\in R_{a}}\text{exp}\left(o_{h}\beta \right)}\right\}-\frac{\left[\sum_{h\in R_{a}}o_{hr}\text{exp}\left(o_{h}\beta \right)\right]\left[\sum_{h\in R_{a}}o_{hs}\text{exp}\left(o_{h}\beta \right)\right]}{{\left[\sum_{h\in R_{a}}\text{exp}\left(o_{h}\beta \right)\right]}^{2}}\left(1\leq r,\;s\leq k\right). \end{array}$$

Weight functions *w(t)* are now introduced, which may be used to weight the contributions to the log partial probability at the *m* uncensored surviving times in various ways. As solutions to weighted scoring calculations $\hat{\beta_{r}}$, balanced maximum likelihood estimations of regression parameters  *β*_*r*_1 ≤ *r* ≤ *k*, are obtained.

(4) $$\begin{array}{c} \sum_{a=1}^{n}w\left(t_{a}\right)\frac{\partial s_{a}}{\partial \beta_{r}}=0. \end{array}$$

The Weights *w(t)* will be dealt. Under proportional risks, the asymptotic characteristics of the weighted estimator have been discussed. While a comprehensive examination of non-proportionality of risks along the lines of Struthers is beyond the scope of this contribution, the findings reveal that the results offered in a technical report corroborate good empirical performance.

## 4. Results and Discussion

The endometrial cancer detection performance is evaluated. Endometrial cancer detection approaches such as immunocystochemistry, genomic analysis, proteome and transcriptome analysis, as well as the suggested multi-cox regression model, are assessed and contrasted. Comparisons of several methods for identifying endometrial cancer are shown in Table 1.

### 4.1. Immunocytochemistry

The immunocytochemistry examinations on the human samples were carried out in the manner previously described. The Ki67 antibody and the antiTRIB3 antibody were the antibodies employed. We also used appropriate negative controls, such as those that had no main antibody. The findings of immunocytochemical staining were analyzed using predefined criteria. The proportion and intensity of staining were taken into account while grading immunostaining. Each sample was given a histology score ranging from 0 to 300.

The formula used to calculate the score was as follows:

(5) $$\begin{array}{c} \text{Histoscore}=1\times \left(\%\text{ light staining}\right)+2\times \left(\%\text{ moderate staining}\right)+3\times \left(\%\text{ strong staining}\right). \end{array}$$

An automated imaging device, the ACIS III Instrument (DAKO), was also employed to assist in the grading of immunocytochemistry. Four separate locations of each sample were used to calculate an intensity score, with values ranging from 60 to 255.

### 4.2. Genome Analysis

The purpose of this work was to discover alternate splicing events in endometrial cancer by whole genome analysis and to create a predictive model for the disease (EC). In order to forecast the prognosis of Endometrial Cancer patients based on PASEs, the researchers used whole genome analysis of Alternative Splicing events to develop their model. This model provides a credible theoretical framework for evaluating the clinical prognosis of Endometrial Cancer patients. The researchers also conducted a whole genome study of PASEs and developed a predictive model for EC. No reports, however, have been published on research that used whole genome analysis to identify alternative splicing events in endometrial cancer.

### 4.3. Transcriptomic Analysis

Total RNA was isolated from endometrial tissue samples utilizing an RNeasy Mini Kit from Qiagen. RNA samples were depleted of ribosomal RNA (rRNA) and processed for sequencing as per the Illumina TruSeqRNA sample preparation instructions, and then submitted to 120 base pair (bp) paired-end sequencing using the Illumina HiSeq2500. They investigated the endometrial cancer proteome utilizing data from the TCGA transcriptomics project as well as antibody-based protein data. Based on transcriptomics data from 541 patients, 1635 genes have been identified as prognostic; 787 genes are related with a poor prognosis, while 848 genes are associated with a positive prognosis. The transcriptome study reveals that 73 percent (*n* = 14572) of all human genes (*n* = 20090) are expressed in endometrial cancer. All genes were grouped into one of the 5 groups dependent on the ratio of mRNA levels in endometrial tumor compared to the mRNA levels in the other 16 tumor tissues studied.

### 4.4. Proteomic Analysis

Proteomics investigation of microdissected cell populations, despite the analytical benefits provided by laser microdissection for cell-type-specific genomic research, has been restricted. A crucial analytical approach of proteomics, nanoLC combined online with tandem mass spectrometry, has traditionally needed many cells than can be easily gathered by laser microdissection in order to provide high-quality proteomic data. Research methodologies that depend on pooling cells from many sections are impracticable when time or tissue sample sizes are limited, such as in fresh or frozen clinical specimens. Endometrial tumor cells were extracted utilizing a laser microdissection (LMD) and proteins were identified using high-resolution mass spectrometry (MS)-dependent proteomic assessment.

### 4.5. Molecular Analysis

Molecular testing can distinguish between individuals with a favorable and worse prognosis after excluding non-endometrioid cancers. Patients with endometrial cancer who are presently categorised as high-risk due to clinicopathological characteristics may have their risk assessed using very simple molecular tests. Four unique prognostic groupings in high-risk endometrial cancer have been identified by molecular research, with treatment implications. Targetable modifications were found in high numbers and might be used to tailor therapy to each patient's needs.

#### 4.5.1. Accuracy

**Figure 2:**
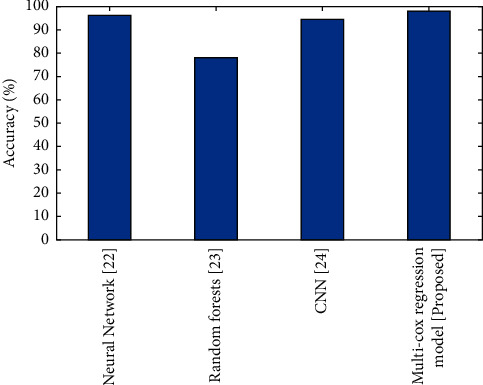
Comparison of Accuracy (%) for existing and proposed methods.

The accuracy refers to the cases that were successfully categorised for the provided test dataset, as shown in Figure 2.

(6) $$\begin{array}{c} \text{Accuracy}=\frac{TP+TN}{TP+TN+FP+FN}. \end{array}$$

Figure 2 presents a comparison of accuracy among the present and suggested procedures for detecting endometrial cancer. The graph clearly demonstrates that the new technique is more accurate than the existing ones. The accuracy of multi-cox regression model is better compared to the CNN, neural network, and random forest.

#### 4.5.2. Specificity

The area under the curve (AUC) for endometrial thickness as a predictive factor of endometrial cancer detection was substantially greater than the AUC for random assignment. The ROC curve, on the other hand, shows that none of the cutoff points produced the best diagnostic findings in terms of integrating greater sensitivity with adequate specificity rates, as required by clinical practise.

(7) $$\begin{array}{c} \text{Specificity}=\frac{TN}{TN+FP}. \end{array}$$

**Figure 3:**
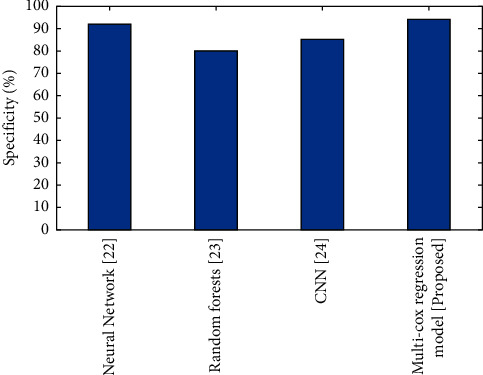
Comparison of Specificity (%) for the existing and proposed method.

The evaluation of specificity for the present and proposed approaches is shown in Figure 3. The specificity of multi-cox regression model is better compared to the CNN, neural network, and random forest.

#### 4.5.3. Sensitivity

Improved diagnostic performance appears to be beneficial for improved risk classification in Endometrial cancer patients. The improved sensitivity is almost certainly due to the inclusion of more texture features in the evaluation.

(8) $$\begin{array}{c} \text{Sensitivity}=\frac{TP}{TP+FN}. \end{array}$$

**Figure 4:**
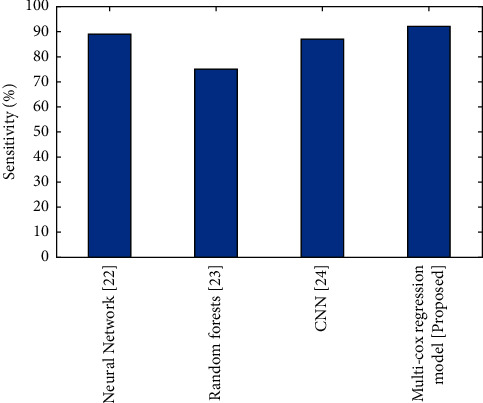
Comparison of Sensitivity (%) for the existing and proposed method.

The evaluation of sensitivity for the present and proposed approaches is shown in Figure 4. The sensitivity of multi-cox regression model is better compared to the CNN, neural network, and random forest.

## 5. Conclusion

Endometrial tumor sufferers might get adjuvant treatments in a variety of ways. Threat factors, molecular classification, particular mutant gene, and metastatic behavior may play a significant role. Although locally advanced Endometrial tumor can now be managed with multimodality therapy. Therapy for metastatic Endometrial Tumor, on the other hand, is still tough. In future medical trials, new agents should be tested along with appropriate molecular biomarkers and stratification of known key prognostic variables. Furthermore, in a non-dramatic approach, adjuvant treatment for initial phase endometrial tumor is being investigated. The usual adjuvant treatment, however, raises a number of problems about its actual advantage and the best way to deliver radiation. Chemotherapy's small benefit and hormonal treatment hazy benefits limit its widespread use. This specific group has a restricted amount of research due to their generally best prognosis. Furthermore, these studies usually include a variety of patient characteristics, illnesses, and surgical therapy. These factors make it difficult to reach a definitive judgement or make a suggestion about the best adjunctive therapy. The researchers investigated the guiding effect of circulating tumor cells and used the multi-cox regression method to detect the initial phase of endometrial tumor and its morphological and biological features, as well as immunocytochemistry, genomic analysis, transcriptomic analysis, proteomic analysis, and molecular analysis. The multi-cox regression method excels existing techniques such as CNN, Random forest, and neural network in terms of accuracy, specificity, and sensitivity.

## Figures and Tables

**Table 1:** Comparison of different Techniques for detecting endometrial cancer.

Techniques	Accuracy (%)	Sensitivity (%)	Specificity (%)
Neural network [22]	96	89	92
Random forest [23]	78	75	80
CNN [24]	94.5	87	85
Multi-cox regression model	97.84	92	94

## Data Availability

The datasets used and/or analyzed during the present study can be available from the corresponding author if needed.
